# Integrative Single‐Cell and Machine Learning Analysis Reveals Immune Microenvironment Remodelling in Lymph Node Metastasis of Lung Adenocarcinoma

**DOI:** 10.1111/jcmm.70859

**Published:** 2025-09-23

**Authors:** Shuai Jiang, Jinhan Zhao, Jiaqi Feng, Yue Yu, Lianmin Zhang, Chenjun Huang, Zhenfa Zhang

**Affiliations:** ^1^ Department of Lung Cancer, Tianjin Lung Cancer Center, National Clinical Research Center for Cancer, Key Laboratory of Cancer Prevention and Therapy, Tianjin's Clinical Research Center for Cancer Tianjin Medical University Cancer Institute and Hospital Tianjin China; ^2^ London International Academy London Ontario Canada; ^3^ Department of Thoracic Surgery The First Affiliated Hospital With Nanjing Medical University Nanjing China

**Keywords:** lung adenocarcinoma, lymph node metastasis, risk prediction model, single‐cell sequencing, tumour microenvironment

## Abstract

Lymph node metastasis is a pivotal determinant of prognosis in lung adenocarcinoma, yet its impact on tumour microenvironment remodelling remains insufficiently characterised. In this study, we employed single‐cell RNA sequencing to compare metastatic and non‐metastatic lymph nodes, delineating metastasis‐associated immune and stromal alterations. Metastatic nodes exhibited marked reductions in dendritic cell and T cell infiltration alongside increases in monocytes and SPP1^+^ macrophages, indicative of an immunosuppressive milieu. Intercellular communication analysis revealed strengthened interactions among SPP1^+^ macrophages, monocytes, and epithelial cells, suggesting coordinated signalling that may further enforce immune suppression. Integrating differentially expressed genes with multi‐omic features, we developed an ensemble machine learning model, LNRScore, which robustly stratified patients into distinct risk groups. A high LNRScore was associated with poorer prognosis and reduced immune infiltration, whereas a low LNRScore correlated with higher immunogenicity and greater predicted responsiveness to immunotherapy based on TCIA assessments. Further analyses identified HMGA1 as a core gene within the model, closely linked to adverse outcomes; functional assays demonstrated that high HMGA1 expression promotes the proliferation and migration of the LLC cell line, supporting its role in metastatic progression. Collectively, this study defines the immune microenvironmental remodelling associated with lymph node metastasis, establishes an effective risk prediction model (LNRScore), and highlights HMGA1 as a potential target for precision diagnosis and therapy in lung adenocarcinoma.

## 1 Introduction

Lung cancer is one of the most common malignant tumours worldwide in terms of both incidence and mortality, with lung adenocarcinoma being the most prevalent histological subtype [1, 2]. Although recent years have seen significant progress in molecular subtyping, targeted therapy, and immunotherapy [3, 4], the overall prognosis for patients with lung adenocarcinoma remains unsatisfactory, especially for those with lymph node metastasis, whose survival time is markedly shortened [5, 6]. As the primary site of distant metastasis in lung adenocarcinoma, lymph nodes serve not only as a ‘bridgehead’ for tumour cell dissemination but also play a vital role in modulating the tumour microenvironment, facilitating immune escape, and mediating drug resistance [7, 8].

The tumour microenvironment (TME), consisting of tumour cells, immune cells, stromal cells, and various cytokines, plays a central role in tumour initiation, progression, and metastasis [9, 10, 11]. Numerous studies in recent years have shown that the remodelling of the TME by tumours not only promotes tumour cell survival and invasion but also accelerates tumour progression through mechanisms such as immune suppression and induction of immune tolerance [12, 13]. Notably, in the unique microenvironment of lymph nodes, complex interactions among tumour cells, local immune cells, and stromal cells lead to the formation of an immunosuppressive milieu, enabling tumour cells to evade immune surveillance and achieve metastatic spread. However, the composition, dynamic changes, and regulatory mechanisms of the microenvironment associated with lymph node metastasis in lung adenocarcinoma remain incompletely elucidated. In recent years, the rapid development of high‐throughput technologies such as single‐cell sequencing has enabled researchers to accurately characterise the cellular composition and functional heterogeneity of the tumour microenvironment at the single‐cell level, providing a solid technical foundation for elucidating the mechanisms of tumour metastasis and immune escape [14, 15, 16]. However, these approaches also bring new analytical challenges, including large data volumes, high complexity, and a multitude of variables, making it difficult for traditional biostatistical methods to fully mine the deep information contained within. In this context, machine learning (ML), as a powerful data‐driven analytical tool, is increasingly becoming a core approach in tumour bioinformatics research [17, 18]. Machine learning methods can efficiently process and integrate multi‐omics data (such as gene expression, mutation profiles, proteomics, etc.), identify potential key molecular biomarkers and predictive factors through feature selection, model integration, and nonlinear analyses, and construct robust and reliable clinical risk prediction models [19, 20]. In recent years, mainstream machine learning algorithms, including random forests, support vector machines, neural networks, and extreme gradient boosting (XGBoost), have been widely used for prognosis evaluation, precision classification, and response prediction in cancer, achieving remarkable results. For lung cancer, risk scoring systems based on multidimensional data integration and machine learning hold great promise for personalised treatment decision‐making.

However, there are still relatively few studies on integrative multi‐omics analysis and the development of highly efficient risk prediction models specifically related to lymph node metastasis in lung adenocarcinoma. How to utilise single‐cell and multi‐omics big data, combined with multiple machine learning algorithms, to accurately identify immune microenvironment changes and molecular features associated with lymph node metastasis—and thereby achieve efficient prediction of metastatic risk, prognosis, and immunotherapeutic response in patients—remains a significant and challenging issue. Therefore, in this study, we employed single‐cell transcriptomics, spatial transcriptomics, and multi‐omics database resources to systematically analyse the remodelling features of the immune microenvironment associated with lymph node metastasis in lung adenocarcinoma, screen for key molecules, and construct a highly efficient and generalisable lymph node metastasis risk prediction model, LNRScore, by integrating multiple mainstream machine learning algorithms. Furthermore, in vitro experiments were conducted to validate the biological functions of the core genes identified in the model, aiming to provide new theoretical insights and methodological tools for in‐depth understanding of lung adenocarcinoma metastasis mechanisms, precise risk stratification, and individualised clinical management.

## 2 Method

### 2.1 Data Resources and Preprocessing Steps

For this research, gene expression profiles, somatic single nucleotide variants (SNVs), and copy number alterations (CNAs) related to lung adenocarcinoma (LUAD) were sourced from The Cancer Genome Atlas (TCGA). To facilitate comparative analyses, gene expression data from normal lung tissues were incorporated using the Genotype‐Tissue Expression (GTEx) project. Significant genomic amplification and deletion regions were identified by applying the GISTIC 2.0 algorithm (https://software.broadinstitute.org) to CNA data. Six separate LUAD transcriptomic datasets—GSE13213 [21], GSE26939 [22], GSE29016 [23], GSE30219 [24], GSE31210 [25], and GSE42127 [26]—were retrieved from the Gene Expression Omnibus (GEO) repository. Batch effects occurring between different cohorts were adjusted through the ComBat normalisation method. Standardised protocols were then followed for subsequent procedures, including data screening, adjustment, and normalisation.

### 2.2 Single‐Cell RNA Sequencing Data Handling

Single‐cell RNA sequencing datasets were procured from the GEO database (accession GSE131907). Initial raw count matrices were processed according to quality control workflows implemented in the Seurat R package [27]. Genes expressed in fewer than 10 cells per sample were excluded from subsequent steps. For cell quality control, those with fewer than 200 or more than 5000 detected genes, as well as cells with mitochondrial RNA reads accounting for over 10% of total UMIs, were discarded. To achieve cross‐sample integration, the Harmony R package was employed. The workflow incorporated the identification of highly variable genes, principal component analysis (PCA), and dimensionality reduction via uniform manifold approximation and projection (UMAP) based on the leading 30 principal components. Marker genes specific to each cell subset were determined using the FindAllMarkers function, while cell populations were annotated according to established lineage markers reported in previous publications.

### 2.3 Intercellular Signal Transduction Profiling

To examine distinctions in cell–cell signalling between normal lymph nodes and those with metastasis, we utilised the CellChat toolkit to model intercellular communication networks from single‐cell RNA sequencing datasets. Normalised gene expression matrices and cell identity annotations for each group were input into CellChat, which then predicted potential ligand–receptor interactions among cell subsets and quantified their communication likelihood and strength. This process enabled comparison of communication quantity and patterns in the two lymph node types. Visualisation of the resulting networks revealed shifts in intercellular signalling pathways related to lymph node metastasis.

### 2.4 Cell State Co‐Occurrence and Subcluster Distribution in Lymph Nodes

To evaluate differences in cell state associations and the distribution of cellular subpopulations between normal and metastatic lymph nodes, we performed cell state co‐occurrence analysis for each lymph node type using single‐cell transcriptomic data. Relative abundances of each subcluster were calculated at the sample level for both groups. Within normal and metastatic lymph nodes, Spearman correlation coefficients were determined between subcluster pairs based on these abundance values, generating correlation matrices that identified coordinated enrichment (positive correlations) or mutual exclusivity (negative correlations) among cellular populations. The full co‐occurrence patterns were visualised with the pheatmap package, enabling direct comparison of network structures between the two lymph node types. For a quantitative assessment of differences in subcluster enrichment, we calculated the observed‐to‐expected (Ro/e) ratio for each subcluster in normal and metastatic lymph nodes. Expected cell numbers were estimated under a random distribution model using chi‐square analysis. Observed frequencies were normalised to the expected values, with Ro/e > 1 indicating specific enrichment in that group. Statistical significance was determined by the chi‐square test (*p* < 0.05).

### 2.5 Development and Validation of the LNRScore Model

Single‐cell RNA sequencing data from lymph nodes were used to cluster and identify epithelial cell populations associated with lung adenocarcinoma metastasis. Data preprocessing and integration were performed using the Seurat package, and epithelial subclusters were classified as metastatic or non‐metastatic. Differential expression analysis between these groups was conducted with the FindMarkers function. Candidate marker genes were further validated in TCGA and independent GEO bulk RNA‐seq cohorts. For the development of the lung adenocarcinoma lymph node metastasis risk score model (LNRScore), differentially expressed genes were subjected to univariate Cox regression to identify those linked to prognosis. Feature selection and model construction incorporated LASSO, Ridge regression, random survival forest (RSF), CoxBoost, and elastic net methods within a 10‐fold cross‐validation framework. The final LNRScore was established based on the selected genes and their risk coefficients and applied to both the training and validation datasets, dividing patients into high‐ and low‐risk groups according to the median score. The predictive performance and robustness of the LNRScore were comprehensively evaluated in the TCGA cohort and multiple independent GEO datasets using various statistical metrics, including the concordance index (C‐index), time‐dependent AUC, and Kaplan–Meier survival analysis, to rigorously assess its prognostic value and generalisability.

### 2.6 Immune Landscape Assessment

The immunophenoscore (IPS) for LUAD patients was obtained from The Cancer Immunome Atlas (TCIA) to estimate their likelihood of benefiting from immunotherapy [28]. Additionally, immune cell infiltration levels in the TCGA cohort were analysed using data from the TIMER2.0 database [29], which synthesises multiple analytical methods to provide an in‐depth view of the tumour immune microenvironment.

### 2.7 HMGA1 Knockdown and Functional Assays in LLC Cells

Murine lung cancer LLC cells were cultured in RPMI 1640 medium with 10% fetal bovine serum and 1% penicillin–streptomycin at 37°C with 5% CO_2_. For HMGA1 knockdown, cells were transduced with lentiviral shRNA targeting HMGA1, and stable clones were established after drug selection. Knockdown efficiency was verified by quantitative RT‐PCR. For migration assays, equal numbers of HMGA1‐knockdown and control LLC cells were seeded into the upper chamber of transwell inserts. After incubation, cells migrating to the lower surface were fixed, stained, and quantified under a microscope. For the colony formation assay, cells were seeded in 6‐well plates at low density and cultured for 10–14 days. Colonies were then fixed, stained with crystal violet, and counted to assess clonogenic capacity. All assays were performed in triplicate.

### 2.8 Statistical Methods

Statistical analyses were conducted using R software (version 4.2.0). For comparisons between two groups, unpaired Student's *t*‐test was applied to normally distributed data, whereas the Mann–Whitney *U* test was used for non‐parametric datasets. Multiple group comparisons were carried out using one‐way ANOVA with Tukey's post hoc test for parametric data, or the Kruskal–Wallis test followed by Dunn's multiple comparisons for non‐parametric variables. Associations between variables were assessed with Pearson's correlation analysis. Data are expressed as mean ± standard deviation (SD), and statistical significance was set at *p* < 0.05. Significant differences are indicated as follows: *p < 0.05, **p < 0.01, ***p < 0.001.

## 3 Result

### 3.1 Single‐Cell Transcriptomic Analysis Reveals Remodelling of the Immune Microenvironment Driven by Lymph Node Metastasis

**FIGURE 1:**
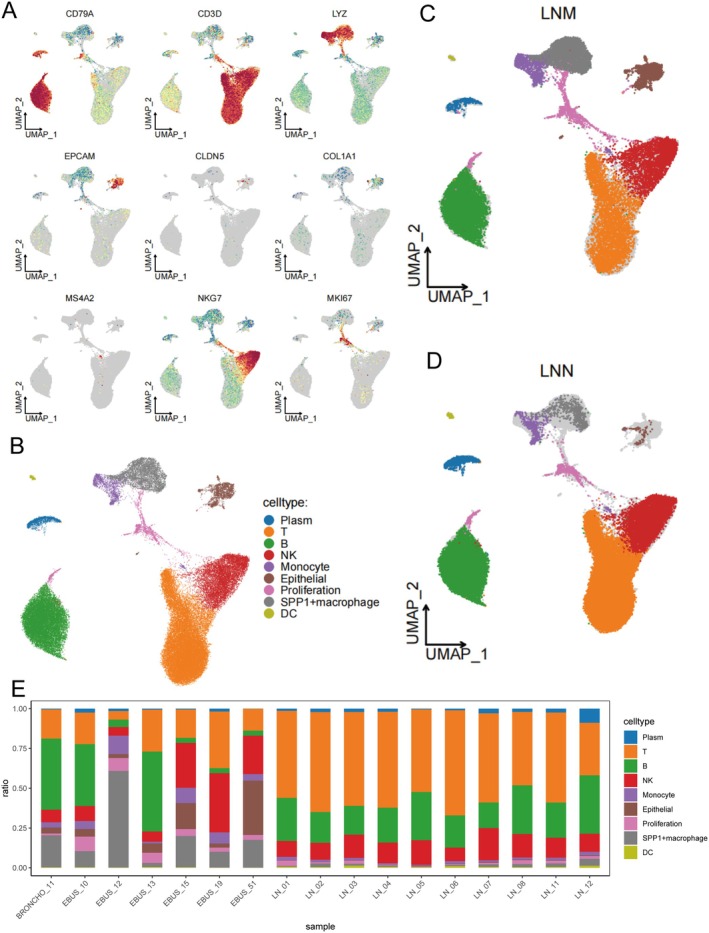
Single‐cell RNA sequencing reveals the cellular composition and immune landscape of lymph node samples in lung adenocarcinoma. (A) UMAP plots showing the expression distribution of nine classical marker genes (CD79A, CD3D, LYZ, EPCAM, CLDN5, COL1A1, MS4A2, NKG7, MKI67) in lymph node‐derived single cells. Typical cell populations including B cells, T cells, monocytes/macrophages, epithelial cells, endothelial cells, fibroblasts, NK cells, and proliferating cells were clearly identified. Notably, the mast cell marker MS4A2 showed almost no positive population, indicating the absence or scarcity of mast cells in lymph node tissue. (B) UMAP plot of all analysed lymph node cells, annotated by cell type based on canonical marker gene expression. (C, D) UMAP projections illustrating the cellular landscape of metastatic (LNM, C) and non‐metastatic (LNN, D) lymph node samples, revealing differential abundance of immune cell subsets. (E) Bar plot showing the proportion of each annotated cell type in individual lymph node samples, highlighting the inter‐sample heterogeneity and the distinct distribution of cell populations between LNM and LNN groups.

To comprehensively explore the cellular heterogeneity of lymph nodes in lung adenocarcinoma and its alteration during metastasis, we extracted lymph node samples from the GSE131907 single‐cell RNA sequencing dataset for unbiased clustering analysis (Figure 1A–E). Firstly, unsupervised clustering and UMAP dimensionality reduction successfully delineated distinct cellular populations within the lymph node microenvironment. We visualised nine classic marker genes—CD79A, CD3D, LYZ, EPCAM, CLDN5, COL1A1, MS4A2, NKG7, and MKI67—to identify main immune and stromal cell types (Figure 1A). CD79A labelled B cells, CD3D for T cells, LYZ indicated monocytes/macrophages, EPCAM and CLDN5 marked epithelial and endothelial cells respectively, COL1A1 identified fibroblasts, NKG7 highlighted NK cells, and MKI67 denoted proliferating cells. Notably, the mast cell marker MS4A2 showed almost no positive cell cluster in the lymph node samples, indicating that mast cells were absent or extremely rare in this microenvironment—a phenomenon that may represent a unique feature of lymph node tissue. Next, based on these canonical markers, the cellular clusters were annotated into eight main populations: plasma cells, T cells, B cells, NK cells, monocytes, epithelial cells, proliferating cells, SPP1^+^ macrophages, and dendritic cells (Figure 1B). Mast cells were not evident in the dataset. To investigate the impact of lymph node metastasis, we further stratified the dataset according to lymph node metastatic status. In the metastatic (LNM) group, UMAP analysis (Figure 1C) revealed a significantly lower proportion of dendritic cells and T cells compared to the lymph node‐negative (LNN) group (Figure 1D). In contrast, LNM samples exhibited marked enrichment for monocytes and SPP1^+^ macrophages, cell types known to be associated with immunosuppression. These data suggest that lymph node metastasis is linked to a shift toward an immunosuppressive cellular environment, characterised by increased myeloid and suppressive macrophage populations and reduced adaptive immune infiltration. Finally, we quantified the proportion of each annotated cell type in each sample (Figure 1E). The bar plot illustrated sample‐level heterogeneity but consistently showed that LNM samples had a higher abundance of monocytes and SPP1^+^ macrophages and fewer dendritic cells and T cells compared to LNN samples. In summary, our single‐cell transcriptomic analysis demonstrates that lymph node metastasis in lung adenocarcinoma induces a profound remodelling of the immune microenvironment. This includes a shift from adaptive immune cells toward immunosuppressive myeloid and macrophage populations, which may promote immune evasion and tumour progression.

### 3.2 Cellular Composition and Correlation Analysis in Metastatic and Non‐Metastatic Lymph Nodes

**FIGURE 2:**
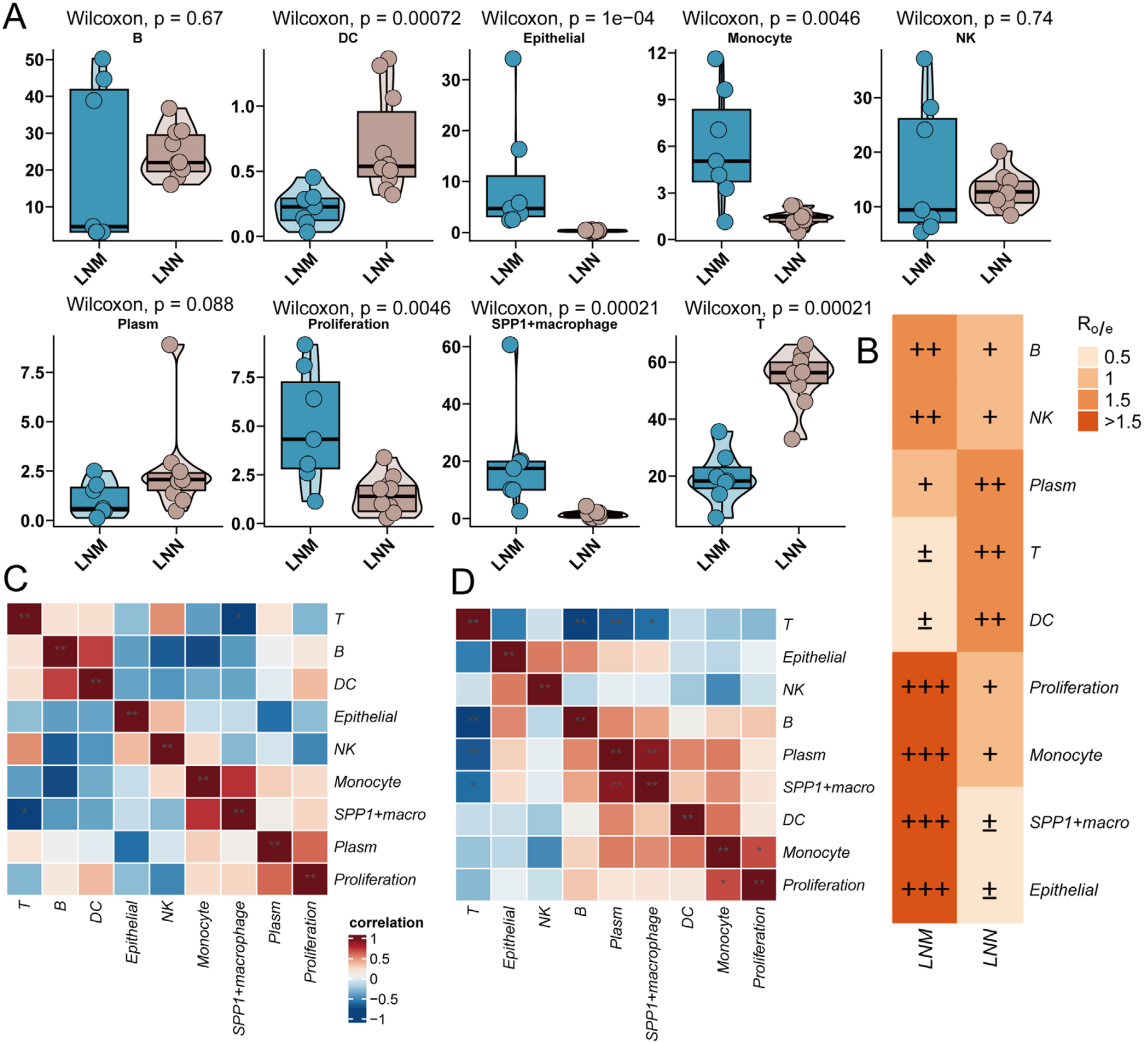
Cellular composition and correlation analysis in metastatic and non‐metastatic lymph nodes. (A) Proportions of major cell populations in metastatic (LNM) and non‐metastatic (LNN) lymph nodes. Each dot represents the percentage of a given cell type in one sample; horizontal lines indicate the median and interquartile range. Between‐group differences were assessed using the Wilcoxon rank‐sum test. (B) Relative enrichment (Ro/e, observed‐to‐expected ratio) of each cell type in LNM versus LNN. Ro/e > 1 denotes relative enrichment, whereas Ro/e < 1 indicates relative de‐enrichment (i.e., under‐representation relative to the expected level). The number of ‘+’ or ‘−’ symbols qualitatively reflects the magnitude of enrichment or de‐enrichment. (C, D) Correlation heatmaps of major cell types in (C) LNM and (D) LNN groups. Colour encodes Spearman correlation: Strong positive correlations are shown in red, whereas weaker or negative correlations tend toward blue; deeper colours indicate stronger relationships. Notably, SPP1^+^ macrophages and monocytes exhibit a strong positive correlation in LNM (C), which is attenuated in LNN (D).

To comprehensively compare the cellular landscape and intercellular relationships between metastatic (LNM) and non‐metastatic (LNN) lymph nodes in lung adenocarcinoma, we systematically analysed the proportions, relative enrichment, and correlation patterns of major cell populations (Figure 2). First, Figure 2A shows the distribution of key cell types across LNM and LNN groups. Statistical analysis revealed that SPP1^+^ macrophages, monocytes, epithelial cells, and proliferating cells were significantly enriched in the LNM group compared to LNN (Wilcoxon test, *p* = 0.00021, 0.0046, 1e−4, and 0.0046, respectively). In contrast, the proportions of T cells and dendritic cells (DCs) were significantly reduced in the LNM group (*p* = 0.00021 and 0.00072, respectively). There were no significant differences for B cells, NK cells, and plasma cells between the two groups (all *p* > 0.05). These findings demonstrate a shift toward myeloid and tumour‐associated cells, with a concomitant reduction of adaptive immune cells in the metastatic microenvironment. Next, to quantitatively assess the specific enrichment of each cell type, we calculated the Ro/e (Observed/Expected ratio) for each population in LNM versus LNN (Figure 2B). Results showed that SPP1^+^ macrophages, monocytes, epithelial cells, and proliferating cells were markedly enriched in the LNM group (Ro/e > 1), while T cells and dendritic cells were relatively enriched in the LNN group. These data further confirm that metastatic lymph nodes develop an immunosuppressive and tumour‐supportive niche. Finally, the correlation heatmaps (Figure 2C,D) illustrate the relationship patterns across cell types in LNM and LNN, respectively. In the LNM group (Figure 2C), SPP1^+^ macrophages exhibited a strong positive correlation with monocytes, suggesting close coordination or functional interplay between these myeloid populations in the metastatic microenvironment. These two myeloid cell types also showed positive correlations with proliferating cells, but negative correlations with T cells and dendritic cells, indicating a reciprocal relationship between myeloid cells and adaptive immune cells. In contrast, in the LNN group (Figure 2D), the positive correlation between SPP1^+^ macrophages and monocytes was substantially weakened, and the overall correlation pattern among major cell types was weaker and more balanced. This suggests that, in the absence of metastasis, the lymph node microenvironment maintains a more equilibrated cellular network, without dominant cell‐type interplay. In summary, lymph node metastasis in lung adenocarcinoma drives a cell composition shift from adaptive immunity dominance to a myeloid‐ and tumour‐associated cell–enriched state, significantly enhancing the inter‐correlation among myeloid cells and promoting the formation of an immunosuppressive cellular network. These cellular dynamics provide important clues for understanding tumour immune escape and metastatic progression.

### 3.3 Enhanced Cell–Cell Communication Networks in Metastatic Lymph Nodes

**FIGURE 3:**
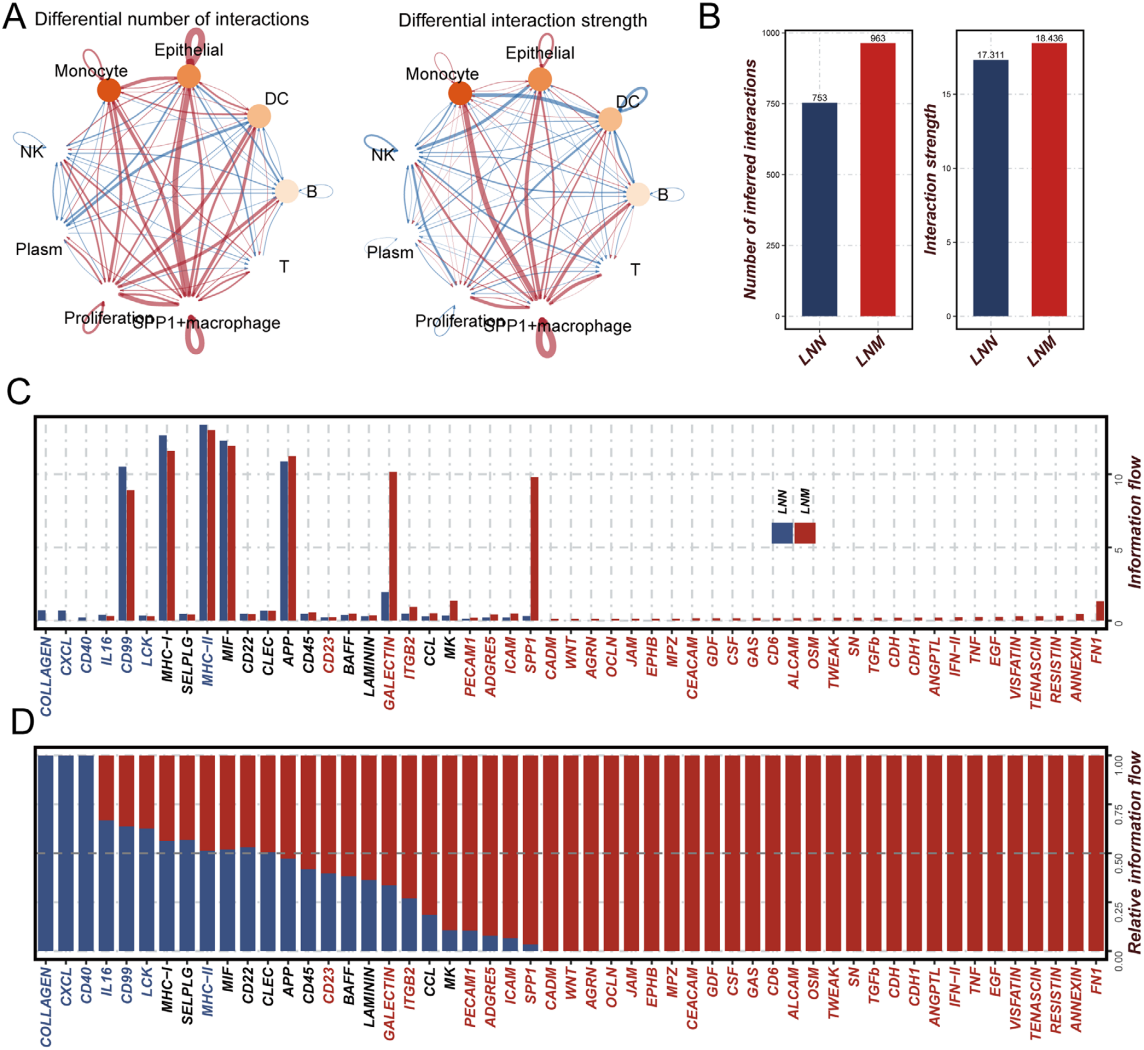
Global rewiring of cell–cell communication networks in metastatic (LNM) and non‐metastatic (LNN) lymph node microenvironments. (A) Cell–cell interaction networks among major cell populations in LNN (left) and LNM (right) samples, coloured by cell type. The thickness and intensity of edges indicate the number and strength of inferred communication events, with red edges highlighting interactions strengthened in LNM. (B) Quantification of the total number of cell–cell interactions and the overall interaction strength in LNN and LNM groups, demonstrating a globally intensified intercellular communication in LNM. (C, D) Heatmaps comparing the information flow of representative signalling pathways in LNN versus LNM. SPP1 and FN1 axes are markedly upregulated in LNM, while COLLAGEN and CXCL signalling are more prominent in LNN, reflecting dynamic pathway remodelling during lymph node metastasis.

To further elucidate the changes in cell–cell communication associated with lymph node metastasis, we systematically analysed the intercellular interaction networks and key signalling pathways in metastatic (LNM) and non‐metastatic (LNN) lymph node microenvironments (Figures 3 and S1). Network analysis (Figure 3A) revealed that in the LNM group, SPP1^+^ macrophages, monocytes, and epithelial cells formed a densely connected network with significantly strengthened interactions, as indicated by the prominent red edges representing enhanced communication in metastatic contexts. Quantitative assessment (Figure 3B) showed that both the number of inferred interactions and the total interaction strength were markedly higher in the LNM group compared to the LNN group (number of interactions: 963 vs. 753; interaction strength: 18,436 vs. 17,311), suggesting that metastasis is accompanied by globally intensified intercellular communications. Analysis of outgoing and incoming interaction strengths for each cell type (Figure S1) further demonstrated that in the metastatic state, SPP1^+^ macrophages occupied a central hub position with dramatically increased outgoing and incoming interaction strengths and the largest number of inferred interactions, highlighting their dominant role in orchestrating the cellular interactome of metastatic lymph nodes. In contrast, in LNN, SPP1^+^ macrophages and other cell types such as monocytes and epithelial cells played a relatively less prominent communicative role. Pathway analysis (Figure 3C,D) showed that the SPP1 and FN1 axes exhibited markedly increased information flow in LNM, indicating their dominance in the metastatic microenvironment. Prior studies have demonstrated that SPP1 interacts with CD44 to promote tumour cell proliferation and metastasis [30], while FN1, through integrin‐mediated adhesion signalling, enhances cell survival and invasion and drives metastatic progression [31]. In contrast, COLLAGEN and CXCL pathways were relatively enriched in LNN, potentially reflecting roles in maintaining local immune homeostasis. Taken together, lymph node metastasis not only strengthens communication between myeloid and epithelial compartments but also remodels the interaction network by activating key pro‐metastatic signalling pathways, and identifies SPP1+ macrophages as critical communication hubs within the metastatic niche.

### 3.4 Integrated Genomic, Transcriptomic, and Prognostic Analysis Identifies Key Genes Associated With Lymph Node Metastasis

**FIGURE 4:**
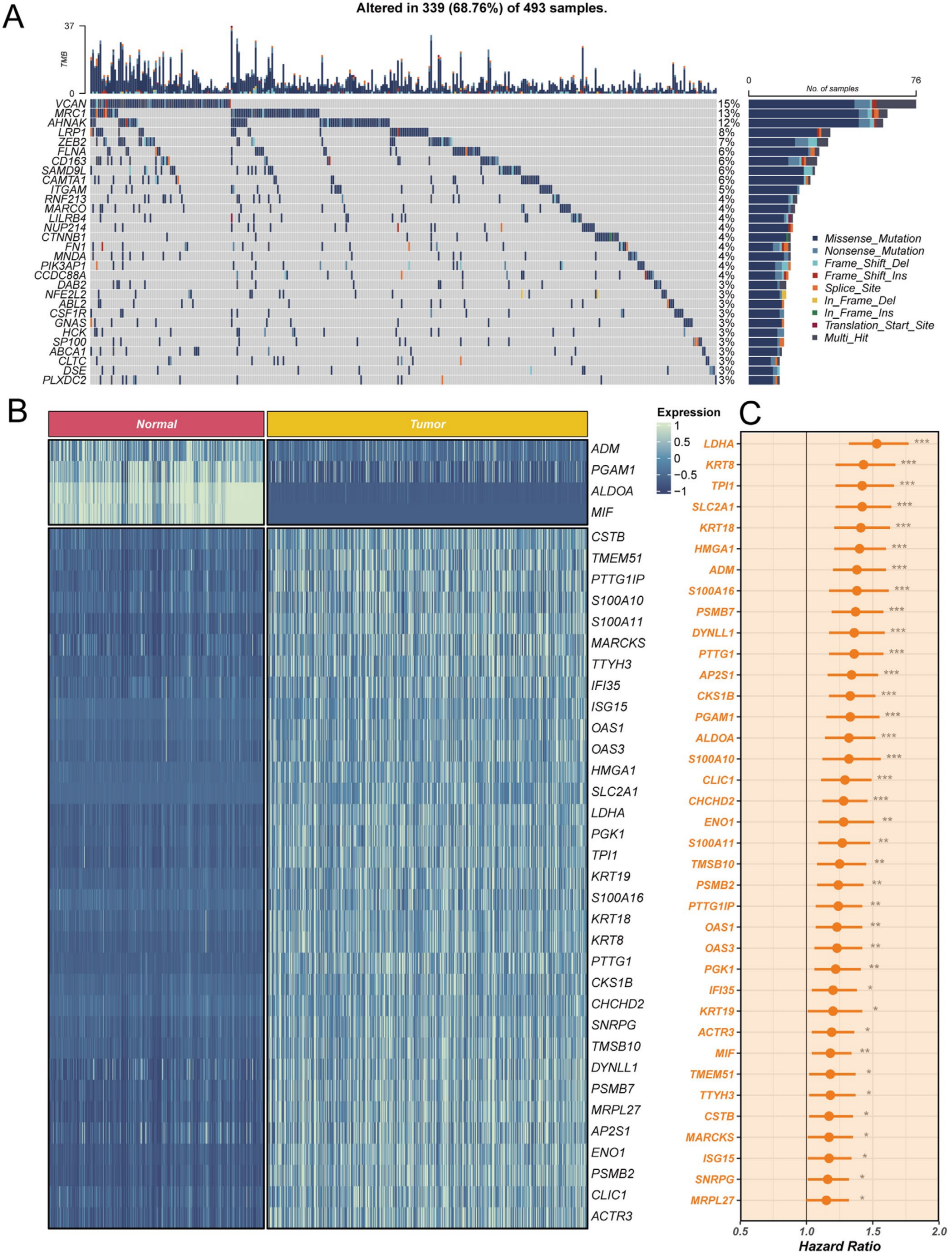
Genomic alteration landscape, differential expression, and prognostic analysis of highly expressed lymph node metastasis (LNM) signatures. (A) Mutation spectrum of the top 30 most frequently mutated LNM‐upregulated genes in TCGA tumour samples. Different colours indicate respective mutation types. (B) Heatmap showing expression of these LNM‐high genes in TCGA tumour versus GTEx normal samples. Left panel: Normal tissues; right panel: Tumour tissues; colour represents gene expression levels. (C) Prognostic value of LNM‐high genes evaluated by Cox regression analysis for overall survival. Orange dots represent high‐risk genes, and asterisks indicate statistical significance.

To elucidate the molecular characteristics associated with lymph node metastasis (LNM), we performed a comprehensive multidimensional analysis of genes highly expressed in LNM (see Figures 4, S2, and S3). First, based on single‐cell differential expression analysis, we identified genes that were upregulated in LNM compared to LNN and systematically evaluated their mutational profiles in TCGA tumour samples. Figures 4A and S2 illustrate the major types and patterns of genetic alterations for these LNM‐associated genes, including variant classification, mutation types, single nucleotide variant (SNV) classes, the number of mutations per sample, and the frequency distribution of commonly mutated genes. The results indicate that missense mutations are the most prevalent variant type, SNPs represent the dominant mutation form, and C>A substitutions are the most frequent SNV class. The median number of mutations per sample was four, suggesting that a substantial proportion of patients harbour multiple mutations in these candidate genes. Among the highly mutated genes, VCAN had the highest mutation frequency (15%), followed by MRC1 and AHNAK. To further validate the robustness of data integration and investigate the genomic distribution of these LNM‐associated genes, we performed principal component analysis (PCA) on seven integrated lung adenocarcinoma cohorts after batch effect removal (Figure S3A). The results revealed a uniform distribution of samples from different cohorts, indicating effective elimination of batch effects. Subsequently, we compared the expression levels of these genes between TCGA tumour samples and GTEx normal tissues (Figure 4B). The results showed that most genes were significantly upregulated in tumour tissues, supporting their potential functional roles in tumorigenesis and metastasis. We then mapped the chromosomal locations of these differentially expressed genes (Figure S3B), demonstrating that tumour‐upregulated genes were mainly enriched in specific chromosomal regions. Further analysis of copy number variations (Figure S3C) revealed that many candidate genes frequently exhibited amplifications or deletions, suggesting that dosage alterations may be closely related to their aberrant expression in LNM. Finally, Cox regression analysis (Figure 4C) showed that high expression of the majority of LNM‐associated genes was significantly associated with poor overall survival, with many representing high‐risk prognostic factors. In summary, LNM highly expressed genes not only exhibit extensive genomic alterations (Figure S2), but are also widely distributed across chromosomes and demonstrate recurrent copy number changes (Figure S3), are significantly upregulated in tumour tissues (Figure 4B), and are strongly correlated with poor prognosis in patients (Figure 4C). These findings underscore the biological and clinical significance of these genes in the process of lymph node metastasis and highlight their potential utility in cancer management.

### 3.5 Development and Validation of a Lung Adenocarcinoma Lymph Node Metastasis Risk Score Model (LNRScore)

**FIGURE 5:**
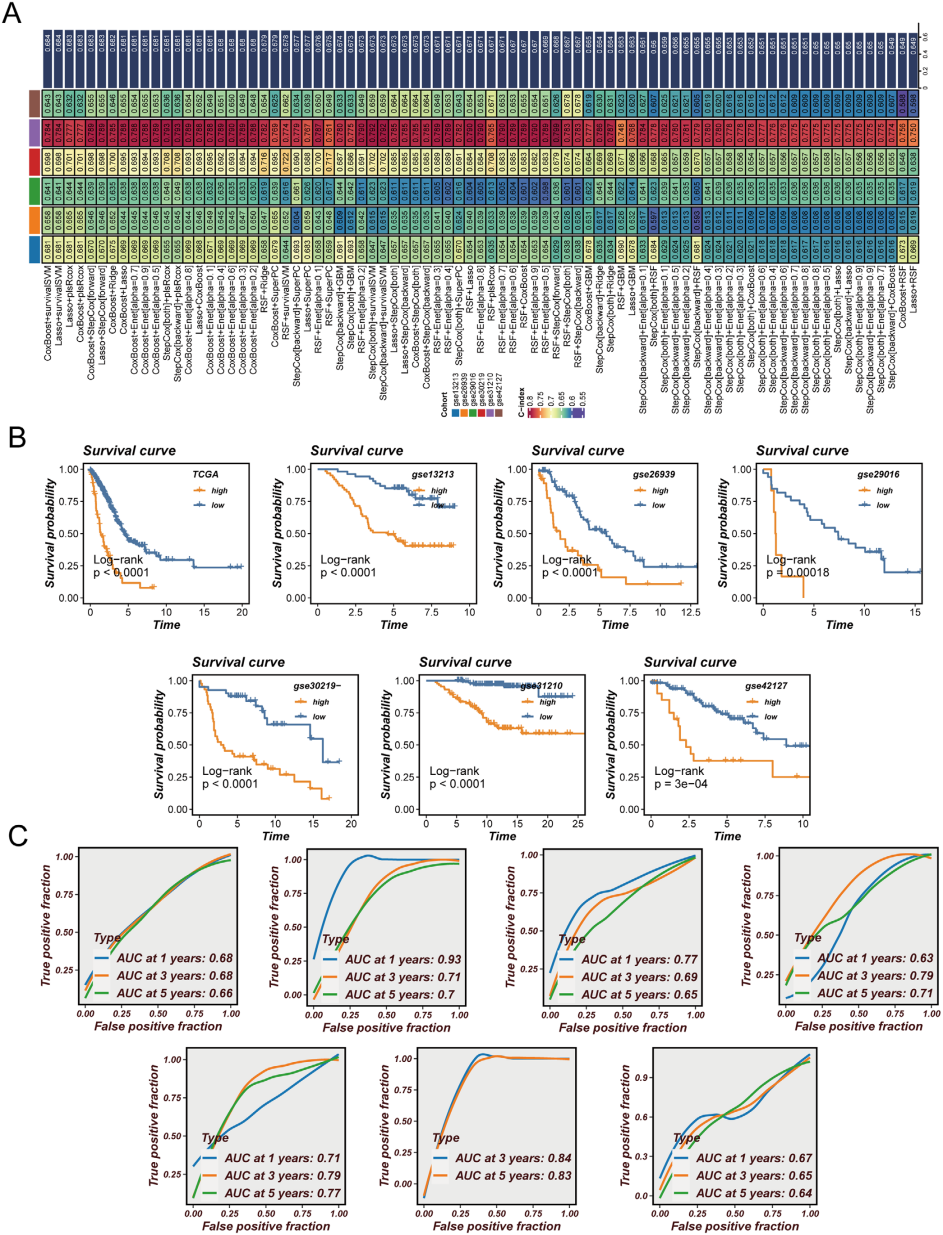
Development and validation of the LNRScore prognostic model for lung adenocarcinoma patients with lymph node metastasis. (A) Schematic overview of the model construction process: Ten mainstream machine learning algorithms and their combinations were systematically evaluated in the TCGA training cohort and six independent GEO validation cohorts, leading to the selection of the optimal integrative model (LNRScore) based on average C‐index values. (B) Kaplan–Meier survival curves for high and low LNRScore groups in the TCGA and all six GEO validation cohorts; patients in the high LNRScore group consistently exhibited significantly worse overall survival (log‐rank test, all *p* < 0.001). (C) Time‐dependent receiver operating characteristic (ROC) curve analyses displaying the predictive performance of the LNRScore model for 1‐, 3‐, and 5‐year overall survival across all cohorts; area under the curve (AUC) values indicate robust sensitivity and specificity of the model.

Based on the identified prognostic genes, we employed 10 mainstream machine learning methods and their various combinations to systematically construct and evaluate multiple prognostic models in the TCGA training cohort and six independent GEO validation cohorts (Figure 5A). By comparing the C‐index values of each model across all cohorts, we ultimately selected the optimal model with the highest average C‐index, which we termed the ‘lung adenocarcinoma lymph node metastasis risk score model’ (LNRScore). Patients in the TCGA and all six external GEO cohorts were stratified into high LNRScore and low LNRScore groups according to this risk score. Survival analyses demonstrated that patients in the high LNRScore group exhibited significantly poorer overall survival compared to those in the low LNRScore group (Figure 5B, log‐rank test, all *p* < 0.001), indicating that LNRScore robustly distinguishes prognostic risk across diverse populations. Furthermore, receiver operating characteristic (ROC) curve analyses were performed to assess the predictive accuracy of the LNRScore model at 1‐, 3‐, and 5‐year survival time points (Figure 5C). The area under the curve (AUC) values for each cohort were consistently favourable at all time points, with some cohorts exceeding an AUC of 0.80, reflecting the model's high sensitivity and specificity. Taken together, the LNRScore model, developed by integrating multiple machine learning algorithms, demonstrates strong and consistent predictive performance for survival risk stratification in lung adenocarcinoma patients with lymph node metastasis across independent cohorts, highlighting its considerable potential for clinical application.

### 3.6 Distinct Immune Microenvironment Characteristics Between High and Low LNRScore Groups

**FIGURE 6:**
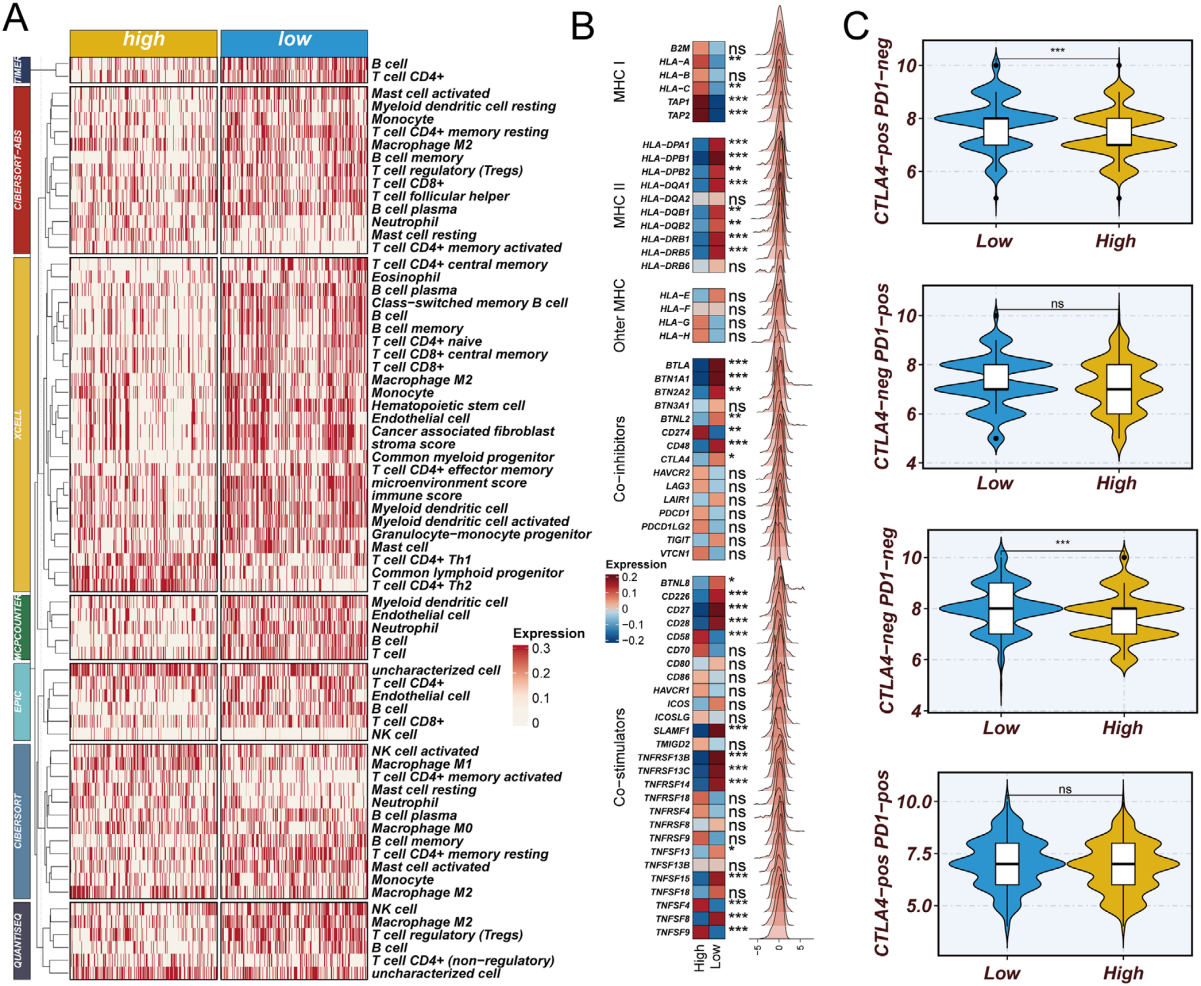
Distinct immune microenvironment characteristics stratified by LNRScore in lung adenocarcinoma. (A) Immune cell infiltration analysis based on the TIMER database reveals significantly higher levels of various immune cells (including CD8+ T cells, CD4+ T cells, B cells, and macrophages) in the low LNRScore group compared to the high LNRScore group. (B) Differential expression analysis of immune‐related genes demonstrates that MHC class II molecules and CTLA4 are significantly upregulated in the low LNRScore group, indicating enhanced antigen presentation and immune regulatory activity. (C) TCIA‐based immune score comparison under different immune checkpoint phenotypes shows that the low LNRScore group has significantly higher immune scores under both CTLA4−/PD1− and CTLA4+/PD1− states, suggesting a potential for increased benefit from immune checkpoint blockade therapies.

To further investigate the association between LNRScore and the tumour immune microenvironment, we systematically analysed immune characteristics between the high and low LNRScore groups. Firstly, immune infiltration analysis based on the TIMER database (Figure 6A) showed that the low LNRScore group exhibited significantly higher infiltration levels of multiple immune cell types—including CD8+ T cells, CD4+ T cells, B cells, and macrophages—compared to the high LNRScore group. This finding suggests that tumours with a low LNRScore are associated with a more active and immunogenic microenvironment. Secondly, the comparison of immune‐related gene expression between the two groups (Figure 6B) revealed that MHC class II molecules and the immune checkpoint gene CTLA4 were significantly upregulated in the low LNRScore group, indicating enhanced antigen presentation and elevated immune regulatory activity. Finally, TCIA database analysis (Figure 6C) demonstrated that the immune score was significantly higher in the low LNRScore group under both the CTLA4−/PD1− and CTLA4+/PD1− phenotypes, implying that these patients may be more responsive to immune checkpoint blockade therapy. No significant difference was observed between groups under the CTLA4−/PD1+ and CTLA4+/PD1+ phenotypes. In summary, patients with low LNRScore are characterised by a more active immune microenvironment and increased expression of immune‐related genes—particularly CTLA4—suggesting they may benefit more from immunotherapy.

### 3.7 HMGA1 Drives Proliferation and Migration

**FIGURE 7:**
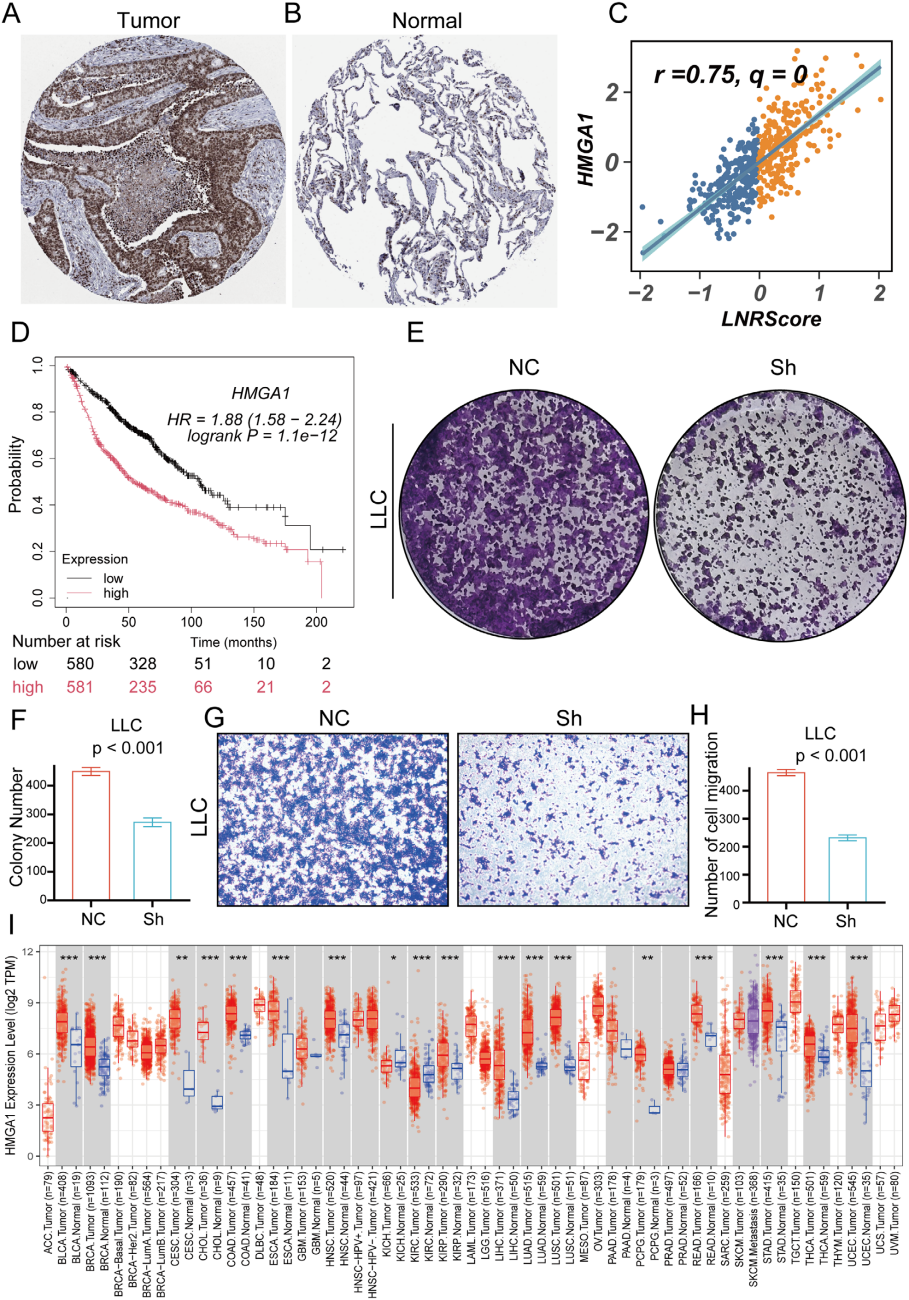
Expression pattern, prognostic value, and functional roles of HMGA1 in lung adenocarcinoma and pan‐cancer. (A, B) Representative immunohistochemical staining images from the HPA database showing high HMGA1 expression in lung adenocarcinoma tumour tissue (A) versus low expression in normal lung tissue (B). (C) Scatter plot depicting a significant positive correlation between HMGA1 expression and LNRScore (*r* = 0.75, *q* = 0). (D) Kaplan–Meier survival analysis (KMplotter) indicating that patients with high HMGA1 expression have significantly worse overall survival than those with low expression. (E, F) Colony formation assays of LLC cells illustrating that HMGA1 knockdown (Sh) markedly reduces colony numbers compared to negative control (NC). (G, H) Transwell migration assays of LLC cells showing suppressed migratory ability following HMGA1 knockdown. (I) Pan‐cancer analysis of HMGA1 mRNA expression (TCGA), demonstrating significantly higher HMGA1 levels in most tumour types compared to matched normal tissues.

To elucidate the role of HMGA1 in lung adenocarcinoma and pan‐cancer, we conducted multi‐dimensional analyses and functional experiments. First, immunohistochemistry images from the HPA database (Figure 7A,B) demonstrated that HMGA1 is highly expressed in tumour tissues compared to normal tissues. Correlation analysis (Figure 7C) further revealed a strong positive association between HMGA1 expression and LNRScore (*r* = 0.75, *q* = 0). Survival analysis using the KMplotter platform (Figure 7D) indicated that patients with high HMGA1 expression exhibited significantly worse overall survival, suggesting its potential as a negative prognostic marker. To further explore its biological function, we performed loss‐of‐function experiments in LLC cells. Colony formation assays showed that HMGA1 knockdown significantly reduced the proliferative capacity of tumour cells (Figure 7E,F). Similarly, transwell migration assays revealed that silencing HMGA1 markedly impaired the migratory ability of LLC cells (Figure 7G,H). Finally, pan‐cancer analysis (Figure 7I) revealed that HMGA1 is significantly upregulated across a broad range of tumour types compared to corresponding normal tissues, highlighting its potential as an oncogenic driver in various malignancies. In summary, HMGA1 expression is positively correlated with LNRScore and poor prognosis in lung adenocarcinoma, promotes tumour cell proliferation and migration, and is widely overexpressed in multiple cancer types, underscoring its potential as a universal oncogenic target.

## 4 Discussion

Lung adenocarcinoma, as the most common subtype of lung cancer, is characterised by a high propensity for lymph node metastasis, which leads to a significantly reduced survival rate [32, 33]. Previous studies have shown that lymph nodes are not only the main route for tumour metastasis but also key regions for immune regulation and microenvironmental remodelling, with their microenvironment tightly linked to metastatic dynamics [10]. However, most traditional research has focused on the tissue or bulk molecular level, making it difficult to reveal changes in cellular subpopulations and their dynamics. In this study, we combined single‐cell sequencing technology to, for the first time, systematically uncover the heterogeneity and immune‐suppressive patterns of the microenvironment in metastatic and non‐metastatic lymph nodes of patients with lung adenocarcinoma, thus providing higher‐resolution evidence for understanding the complex process of lung cancer metastasis.

In recent years, increasing attention has been paid to the complex regulatory functions of the tumour microenvironment (TME), and myeloid‐derived immunosuppressive cells (such as tumour‐associated macrophages and monocytes) are recognised as playing central roles in immune escape and the promotion of tumour metastasis [34, 35]. For example, SPP1^+^ macrophages and other subpopulations have been shown to foster immunosuppressive states in various tumours [36]. However, systematic validation of the cell–cell communication and network structure associated with lymph node metastasis in lung adenocarcinoma has been lacking. Our study not only confirms the aggregation of SPP1^+^ macrophages and monocytes in metastatic lymph nodes, but also uses cell–cell communication mapping to quantitatively demonstrate their enhanced interactions with epithelial cells, revealing the spatial reorganisation of immunosuppressive networks. These findings complement previous descriptive observations and enrich the theoretical framework of ‘spatial network evolution’ in the immune microenvironment. At the molecular regulatory level, previous literature has demonstrated that mutations, copy number variations, and dysregulated expression of tumour‐related genes play critical roles in cancer progression, with some genes even serving as prognostic or subtyping markers [37, 38, 39]. However, a comprehensive landscape of LNM‐specific key molecules, especially their genetic alterations, expression profiles, and prognostic associations, has not been systematically explored. This study integrates single‐cell differential expression, multi‐omics data from large TCGA cohorts, and external validation datasets to systematically identify LNM‐associated mutated genes, their chromosomal distribution, and prognostic value. Especially by performing batch correction and external validation in multicenter and highly heterogeneous samples, the reliability of the results is improved, providing new insights for discovering metastasis‐related molecular targets and understanding microenvironmental evolution. In recent years, machine learning algorithms have been widely used in cancer prognosis evaluation and individualised diagnosis due to their ability to handle complex data and extract high‐dimensional features [40, 41]. However, multi‐omics–integrated predictive models for the risk of LNM in lung adenocarcinoma remain limited. In this study, we innovatively integrated ten mainstream algorithms and established the LNRScore model through large‐scale, multicenter training and external validation, enabling high‐accuracy risk stratification for LNM. Compared with previous models based on a single dimension or low‐dimensional features, LNRScore incorporates molecular, genomic, and immune phenotypic information and is significantly correlated with immune cell infiltration and immunotherapy response, providing an algorithmic foundation for individualised decision‐making and the design of precise immunotherapy regimens.

Our study also focused on the functional mechanisms of key genes in the model, such as HMGA1. Previous reports have indicated that HMGA1 plays an important regulatory role in tumour stemness and metastasis [42, 43], but its specific function in lung adenocarcinoma lymph node metastasis and immunosuppressive networks has not been fully elucidated. Through multi‐level correlation analyses and in vitro functional assays, we validated that high HMGA1 expression not only promotes tumour cell proliferation and migration, but also is directly related to LNRScore and poor immune response, providing more direct experimental evidence for this gene as a novel molecular target.

Naturally, this study has certain limitations. First, although multicenter and multi‐cohort integration and validation were performed, the sample size and tissue types in single‐cell sequencing remain limited, and the functions of some rare cellular subpopulations need further investigation. Second, the model is mainly based on transcriptomic and mutational molecular data; clinical translation will require combination with imaging, pathology, and other multimodal features. Additionally, for key molecules such as HMGA1, further in‐depth investigation is needed to elucidate their upstream and downstream regulatory mechanisms and spatiotemporal dynamics in immune regulation.

## Author Contributions

The study was conceived and designed by Chenjun Huang, Lianmin Zhang, and Zhenfa Zhang. Experiments were performed by Shuai Jiang. Data collection and analysis were performed by Shuai Jiang, Jinhan Zhao, Jiaqi Feng, and Yue Yu. Statistical analysis and data interpretation were carried out by Shuai Jiang and Jinhan Zhao. The manuscript was drafted by Jiaqi Feng, and all authors contributed to manuscript revision. Lianmin Zhang and Zhenfa Zhang supervised the project and are responsible for its overall content as guarantors.

## Ethics Statement

The authors have nothing to report.

## Consent

The authors have nothing to report.

## Conflicts of Interest

The authors declare no conflicts of interest.

## Supporting information


**Figure S1:** Outgoing and incoming interaction strengths of major cell populations in LNN and LNM. Bubble plots showing outgoing (x‐axis) and incoming (y‐axis) interaction strengths for each major cell type in LNN (left) and LNM (right). Bubble size corresponds to the total number of inferred interactions for each cell population. SPP1^+^ macrophages in LNM exhibit dramatically increased outgoing and incoming interaction strengths and are the most interconnected cell population, indicating their central hub role in the metastatic lymph node microenvironment.

**Figure S2:** Integrated characterisation of genomic variants in LNM‐highly expressed genes. (Top left) Variant classification shows missense mutation is predominant.
(Top middle) Variant type distribution indicates SNPs are the most common. (Top right) SNV class frequency with C>A as the most frequent substitution. (Bottom left) Number of variants per sample, median of 4. (Bottom middle) Summary count for each variant classification. (Bottom right) Top 10 most frequently mutated genes and their mutation rates; VCAN shows the highest mutation frequency.

**Figure S3:** Multi‐cohort integrative analysis and chromosomal localization of differentially expressed genes in lung adenocarcinoma. (A) Principal component analysis (PCA) plot of all samples from seven lung adenocarcinoma cohorts after batch effect removal. Samples from different datasets are shown in distinct colours, indicating well‐mixed integration and minimal batch effects. (B) Circos plot illustrating the chromosomal distribution of differentially expressed genes. Red dots represent genes upregulated in tumours, while black dots indicate genes upregulated in normal tissues. (C) Bar plot showing copy number alterations of the differentially expressed genes. Orange bars denote gene amplifications (AMP), and blue bars denote deletions (DEL), with frequencies calculated for each gene and chromosome.

## Data Availability

All data utilised in this investigation is sourced from publicly accessible repositories. The single‐cell transcriptomic data for lymph node samples was extracted from the GSE131907 dataset. Bulk RNA sequencing, somatic mutation, and copy number alteration data were obtained from The Cancer Genome Atlas lung adenocarcinoma cohort (TCGA‐LUAD, https://cancergenome.nih.gov/) and the Genotype‐Tissue Expression Project (GTEx). External validation was performed using independent cohorts from the Gene Expression Omnibus (GEO): GSE13213, GSE26939, GSE29016, GSE30219, GSE31210, and GSE42127.
