# Supplementary figures and images for: Integrative Single‐Cell and Machine Learning Analysis Reveals Immune Microenvironment Remodelling in Lymph Node Metastasis of Lung Adenocarcinoma
Source: J Cell Mol Med. 2025 Sep 23;29(18):e70859. doi: 10.1111/jcmm.70859 (PMC12457218; doi:10.1111/jcmm.70859)

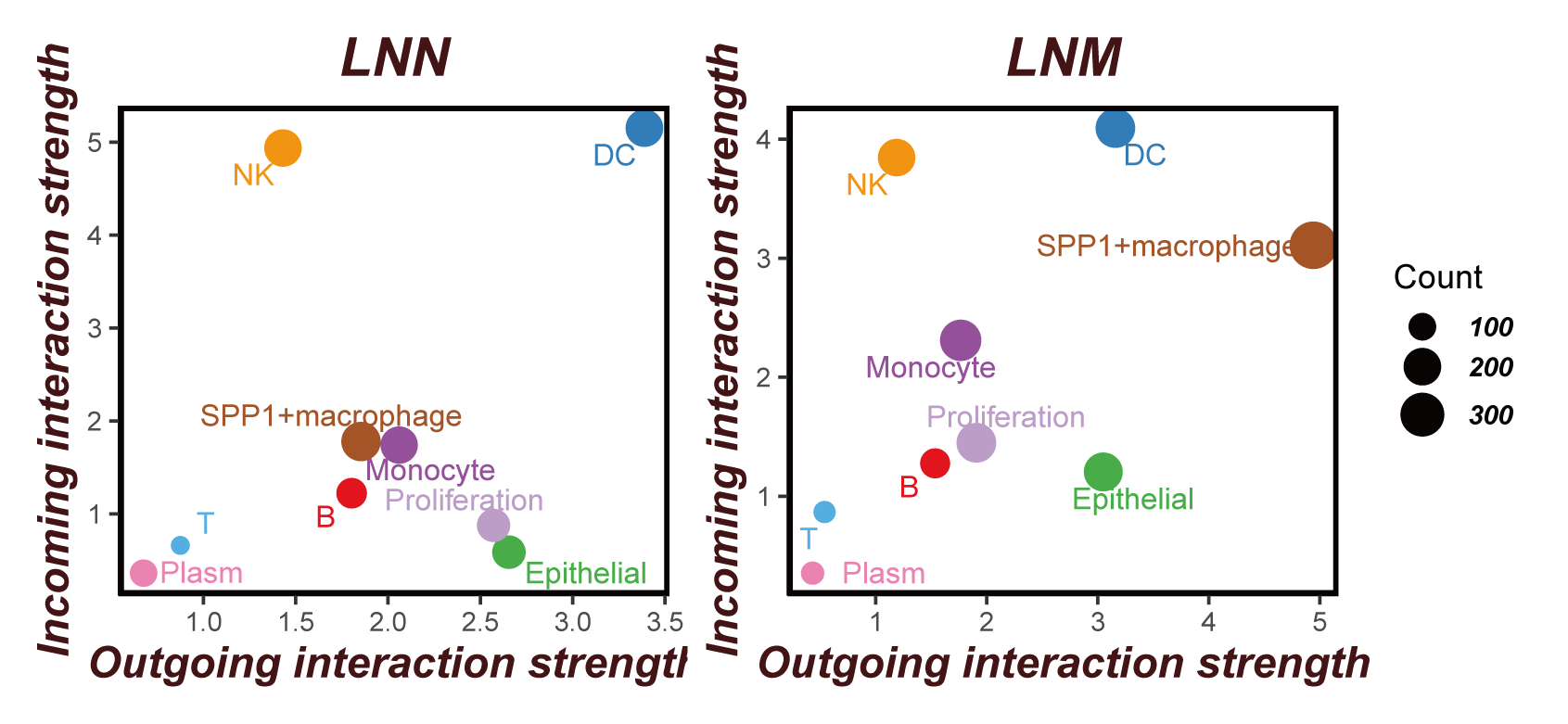

Supplement: Supplementary file 1 — Figure S1: Outgoing and incoming interaction strengths of major cell populations in LNN and LNM. Bubble plots showing outgoing (x‐axis) and incoming (y‐axis) interaction strengths for each major cell type in LNN (left) and LNM (right). Bubble size corresponds to the total number of inferred interactions for each cell population. SPP1+ macrophages in LNM exhibit dramatically increased outgoing and incoming interaction strengths and are the most interconnected cell population, indicating their central hub role in the metastatic lymph node microenvironment. [file JCMM-29-e70859-s002.tif]

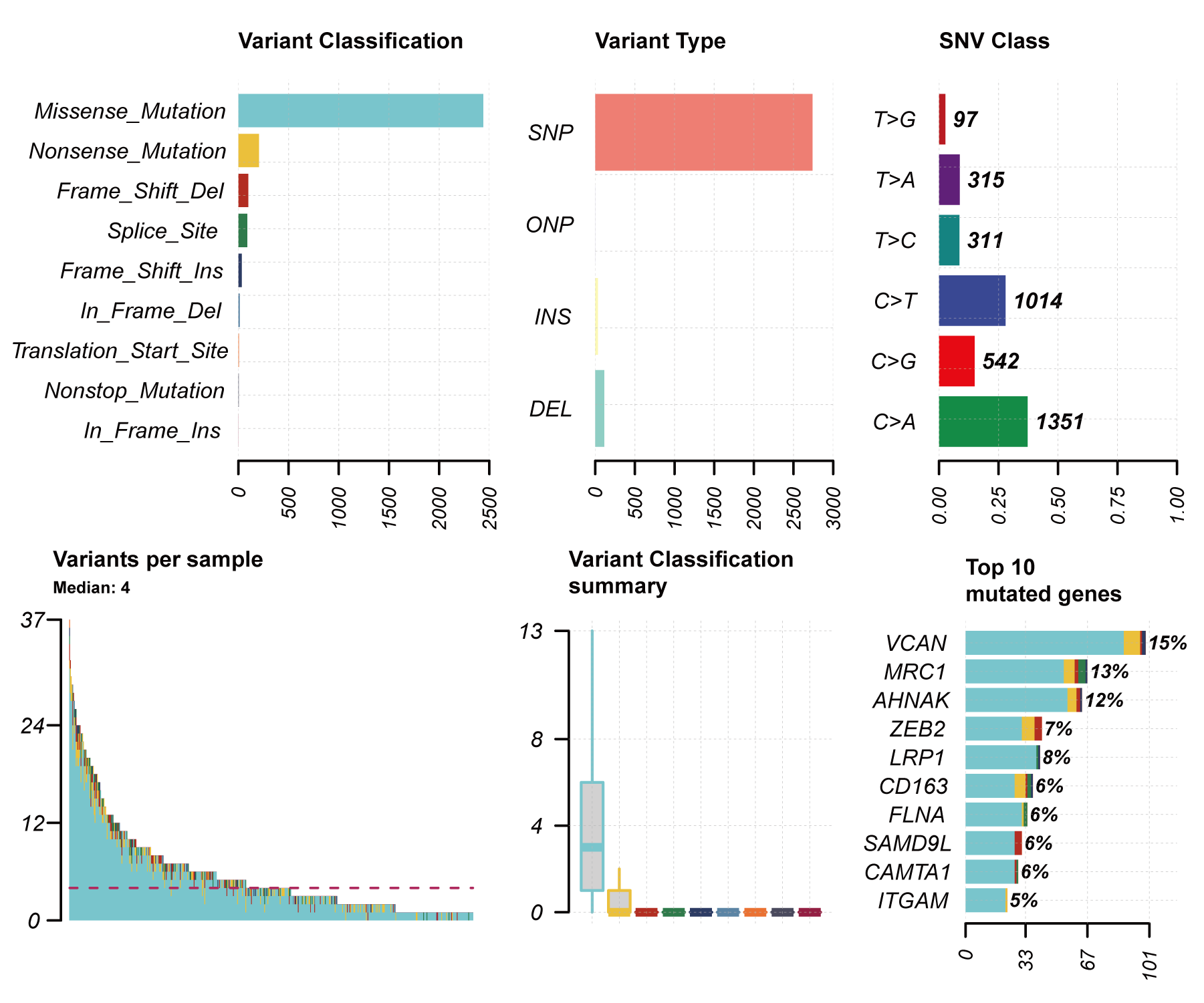

Supplement: Supplementary file 2 — Figure S2: Integrated characterisation of genomic variants in LNM‐highly expressed genes. (Top left) Variant classification shows missense mutation is predominant. (Top middle) Variant type distribution indicates SNPs are the most common. (Top right) SNV class frequency with C>A as the most frequent substitution. (Bottom left) Number of variants per sample, median of 4. (Bottom middle) Summary count for each variant classification. (Bottom right) Top 10 most frequently mutated genes and their mutation rates; VCAN shows the highest mutation frequency. [file JCMM-29-e70859-s001.tif]

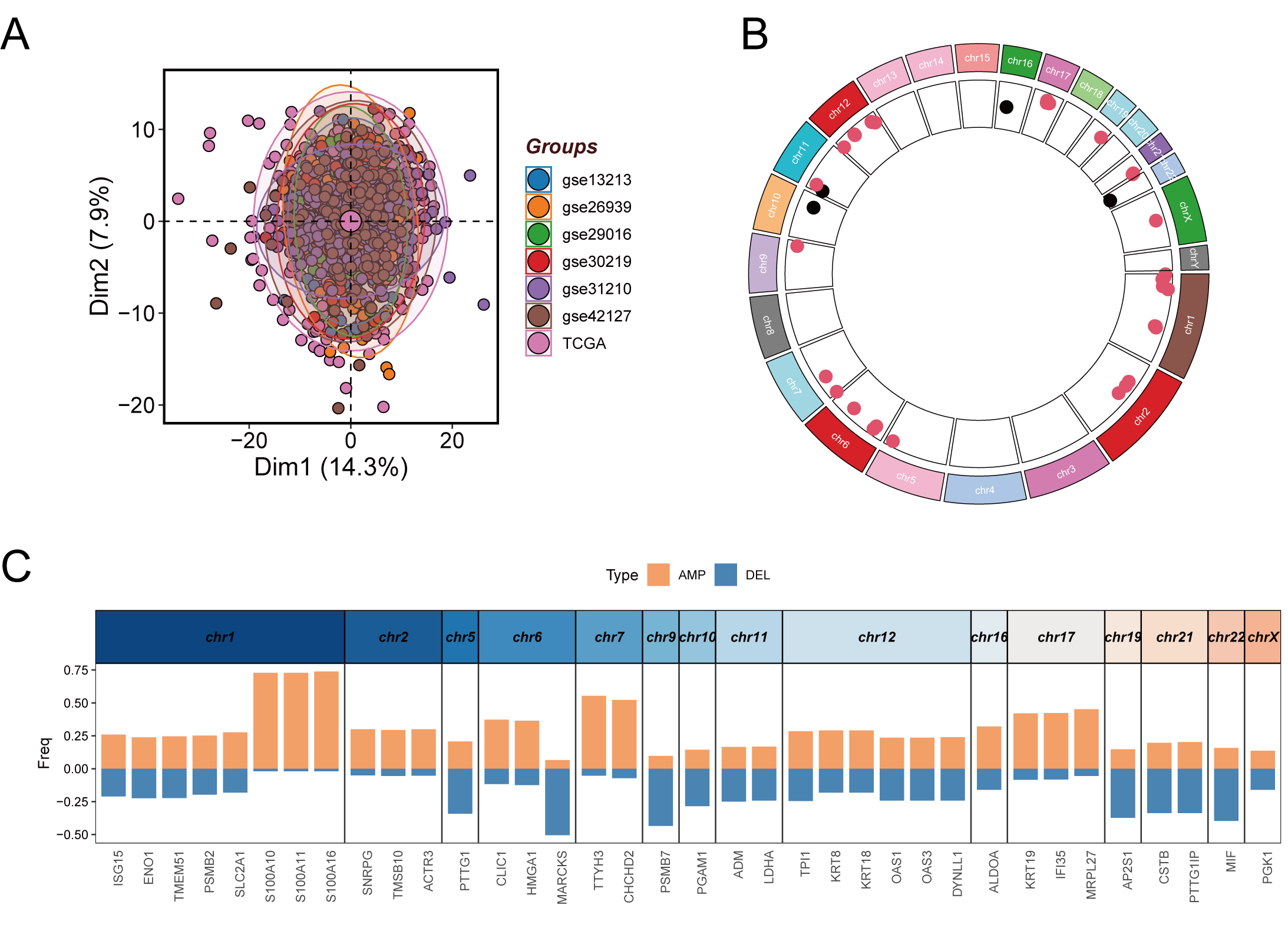

Supplement: Supplementary file 3 — Figure S3: Multi‐cohort integrative analysis and chromosomal localization of differentially expressed genes in lung adenocarcinoma. (A) Principal component analysis (PCA) plot of all samples from seven lung adenocarcinoma cohorts after batch effect removal. Samples from different datasets are shown in distinct colours, indicating well‐mixed integration and minimal batch effects. (B) Circos plot illustrating the chromosomal distribution of differentially expressed genes. Red dots represent genes upregulated in tumours, while black dots indicate genes upregulated in normal tissues. (C) Bar plot showing copy number alterations of the differentially expressed genes. Orange bars denote gene amplifications (AMP), and blue bars denote deletions (DEL), with frequencies calculated for each gene and chromosome. [file JCMM-29-e70859-s003.tif]
